# Disease burden and treatment sequence of polymyositis and dermatomyositis patients in Japan: a real-world evidence study

**DOI:** 10.1007/s10067-021-05939-6

**Published:** 2021-10-22

**Authors:** Celine Miyazaki, Yutaka Ishii, Natalia M. Stelmaszuk

**Affiliations:** 1 Health Economics Department, Janssen Pharmaceutical K.K., Tokyo, Japan; 2 Immunology, Infectious Diseases and Vaccine Department, Medical Affairs Division, Janssen Pharmaceutical K.K., Tokyo, Japan; 3 Real World Evidence Consultant, Parexel International, Stockholm, Sweden

**Keywords:** Dermatomyositis, Healthcare resource utilization, Polymyositis, Treatment choice

## Abstract

**Introduction/objectives:**

Since new consensus on polymyositis (PM) and dermatomyositis (DM) were released in Japan, an updated evidence on treatment landscape and PM/DM burden was essential. This study evaluates treatment burden and overall treatment cost of PM/DM-related inpatient and outpatient visits, treatments, and procedures/patient/year.

**Method:**

This retrospective, observational study analyzed insurance claims from Japan Medical Data Center (JMDC) database. Patients with at least one PM/DM diagnosis/one dispensation of treatment between 1 January 2009 and 31 December 2019 were enrolled. Patient characteristics, treatment patterns and sequence, treatment choices, healthcare resource utilization (HCRU), and related costs were assessed. Chi-square test and linear regression model were used to assess impact of patient characteristics on treatment choice.

**Results:**

Patients (836/4,961) receiving a relevant treatment were analyzed. Heart disease (35%), interstitial lung disease (27%), and diabetes mellitus (26%) were frequently identified as comorbidities. Concomitant dispensation of immunosuppressants and systemic steroids was largely found in first and second line of treatment (LoT) while systemic steroids remained as single dominant treatment across all LoTs. HCRU was very low for inpatient visits (0.68 [1.43]) or rehabilitation (4.74 [14.57]). The mean (SD) number of inpatient visits decreased from first (1.23 [2.32]) to third year (0.11 [0.54]). Total mean (SD) healthcare cost per patients per year was ¥ 3,815,912 (7,412,241), with overall drug dispensation compounding to 80% of total cost.

**Conclusions:**

High concomitant immunosuppressant and systemic steroid prescriptions in first LoT recommend early optimal treatment to manage PM/DM. Although inpatient costs are low, outpatient dispensation costs increase overall economic burden.

**Supplementary Information:**

The online version contains supplementary material available at 10.1007/s10067-021-05939-6.

**Table:** 

**Key Points** *• Japan faces treatment challenges in the prognosis of polymyositis (PM) and dermatomyositis (DM) and thus, we assessed the real-world treatment landscape, practice, patterns, and healthcare resource utilization as an evidence to support healthcare outcome improvement and treatment burden reduction.* *• Systemic steroids were the dominant preferred choice of treatment, but it increases the overall cost of the treatment due to the resultant comorbidities considering possible side-effects promotion.* *• Thus, an increased awareness towards the disease management among patients and medical doctors is required for better management goals based on this real-world practice evidence.*	

## Introduction

Polymyositis (PM) and dermatomyositis (DM) are two subgroups of myositis, also known as idiopathic inflammatory myopathies (IIM), causing chronic inflammation of skeletal muscles and systemic inflammation in skin, lungs, joints, heart, and gastrointestinal tract [1, 2]. They are a group of rare and heterogeneous disorders with a presumed autoimmune pathogenesis [3], identified based on different histopathological features, skin rash, muscular symptoms, and presence of myositis-specific antibodies [1]. Though specific cause of pathogenesis is unknown, environmental factors are believed to influence immune-mediated processes in genetically susceptible individuals which makes early diagnose difficult [4, 5]. Estimated prevalence of PM/DM in Japan was 13.2/100,000 during 2010 and incidence rate was estimated to be 10–13/1,000,000 person-years from 2003 to 2010 [6]. Majority of patients (69%) experienced PM/DM onset during middle age (40–60 years), with females being more susceptible [7]. Japanese Ministry of Health, Labor and Welfare (MHLW) has classified PM/DM as an intractable disease due its unknown etiology, lack of effective treatment, and poor prognosis [8].

Treating PM/DM is challenging due to heterogenicity of disease manifestation, overlapping of clinical symptoms with other autoimmune disorders and myopathies, and need for physical examination and laboratory tests [5, 9]. Current major hurdle in PM/DM treatment is targeted immunotherapy that requires target autoantigens identification, dependable outcome measures due to difference in inclusion criteria among clinical trials which complicates treatment responses assessments [4, 10]. Despite challenges, the treatment focuses on improving patient’s ability to perform daily activities by strengthening muscles and avoid flares and extra-muscular disease in the vital organs [11] for which corticosteroids (GC) with/without immunosuppressants are used as first line of treatment (LoT), and other treatment options include plasmapheresis, intravenous immunoglobulin, and biologic treatments are recommended [12, 13]. Like corticosteroids, treatment refectory patients are prescribed with immunosuppressive drugs that are also not always effective [14].

In 2018, Japan College of Rheumatology, Japanese Society of Neurology, and Japanese Dermatological Association [15] made recommendations and proposed a decision tree for managing PM/DM yet it could not be fully addressed without real-world evidence (RWE) [16]. Multiple studies [6, 7, 17] have provided extensive epidemiological data; however, limited knowledge is available regarding inpatient characteristics and healthcare resource utilization (HCRU). To improve health outcomes and reduce HCRU, it is essential to understand treatment landscape, practice pattern, and HCRU based on insurance claims data. Therefore, the study describes current treatment patterns, clinical characteristics, and disease burden among Japanese PM/DM patients.

## Methods

### Data source

This retrospective, observational, longitudinal cohort study with PM/DM patients analyzed epidemiological health insurance claims data retrieved from Japan Medical Data Center (JMDC) database. The database collects and stores data on inpatient, outpatient, and pharmacy medical claims, as well as demographic data, diagnosis based on ICD-10 codes, drug names and dosages, annual days of therapy, size and types of hospitals, type of claims, and annual company health check-up data and dispensing from multiple health insurance societies and physical examination [18]. The database covered about 5.6 million people in mid-2018.

The data provides prevalence and occurrence rates in general population including healthy people and can track hospital transfers and visits at multiple facilities. Although retirees aged > 65 years are generally not covered by insurance system, a few claims that exist could either be of senior employees/parents of employees covered under family insurance plan.

This study was approved by Rheumatology Research Concept Approval Team in accordance with Japanese ethical and legal guidelines. JMDC data is anonymized electronic health insurance claims data which is created and aggregated under the compliance Personal Information Protection Law Article 2, Paragraph 9. Using this data as secondary research analysis does not require informed consent as per the Ethical Guidelines for Epidemiological Research issued by the Japanese Ministry of Health, Labor, and Welfare (MHLW). This study was designed, implemented, and reported in accordance with the Guidelines for Good Pharmacoepidemiology Practices of the International Society for Pharmacoepidemiology [19] and with the ethical principles laid down in the Declaration of Helsinki [20]. Reporting of claims data was based on International Society for Pharmacoeconomics and Outcomes Research and STORBE guidelines [21].

### Study design and patient population

The inclusion period was 1 January 2009 to 31 December 2019. PM/DM ICD-10 code definition is provided in Appendix 1 Table 3. Index date is the date on which patient received first dispensation of PM/DM treatment. Patients who were diagnosed with PM/DM on/after 1 January 2009 with at least 12 months of follow-up (baseline period), who had a relevant treatment after PM/DM diagnosis, and who had ≥ 1 year of insurance coverage/enrollment before and after inclusion date (date of first PM/DM diagnosis)/index date were included in the study. Follow-up ended on the last day of study (31 December 2019), and date of death/date of lost to follow-up was recorded.

Newly diagnosed patients were identified by lack of PM/DM diagnosis before the index date. Washout period was defined as the period between the first capture (January 2005) of data and first date of PM/DM diagnosis that was recorded not earlier than January 2009. Patients receiving a treatment for PM/DM between 1 January 2005 and 31 December 2008 recorded in the JMDC claims database and those with < 1 year of follow-up or < 12 months before the inclusion date were excluded from the study.

### Study assessments

The primary objective of the study was to describe treatment patterns of PM/DM patients in Japan. The secondary objectives were to describe clinical characteristics and its impact on the choice of treatment, to describe prescriber’s specialty, and to estimate healthcare resource utilization (HCRU) and healthcare cost of PM/DM patients.

#### Characteristics of PM/DM patients

Clinical characteristics of PM/DM patients were defined 12 months prior to the inclusion date/index date. Patient’s age at diagnosis, sex, disease subtype, and comorbidities were assessed during baseline period and age at treatment initiation was assessed before index date. Comorbidities included in the study are reported in Appendix 1 Table 3. Burden of disease was measured using Charlson Comorbidity Index (CCI) score (low [0–2], moderate [3, 4], and high [≥ 5]) and was calculated using a standard CCI algorithm [22]. CCI contains 17 comorbidities, each assigned with a weight (1–6 [0 signifies no diagnosis]) depending upon the risk of mortality associated with the comorbidity. A score of at least one was assigned to a category of a comorbidity if diagnosed 12 months prior to the first PM/DM diagnosis. The prescriber’s specialty for each treatment line was extracted from database based on the first dispensation in the treatment episode. An exploratory analysis was performed to assess the possibility of analyzing prescriber specialty due to high number of missing data.

#### Treatment patterns of PM/DM patients

Time to initiation of a specific PM/DM treatment was defined as the time interval between the date of first PM/DM diagnosis and start date of the treatment of interest. Time to treatment switch was defined as the time interval from initiation of treatment of interest to treatment switch (first treatment had to be discontinued for the switch to appear). Time to add-on was defined as the time interval from the initiation of treatment of interest to the treatment add-on (treatment initiated > 30 days from the start of the first treatment and overlapped with another treatment). Time to treatment discontinuation was defined as the time interval from treatment initiation to discontinuation. The drugs included in this study are given in Appendix 2 Table 4.

If the treatment was < 8 days and did not overlap with another treatment or overlapped but was initiated prior to longer treatment episode, it was not considered treatment line, but as an acute treatment. Concomitant treatment was considered when the second treatment was initiated ≤ 30 days from the start date of first treatment.

#### Sequence of treatment after initiation of first treatment

Sequence of treatment was defined as a switch from first LoT to second LoT, second to third, etc. (dispensation of a different systemic treatment on or after the discontinuation of the previous treatment). A total of nine LoTs were considered. The analysis was performed using episode level information that was defined for each LoT. The start date of a treatment episode was the time of initiation of specific treatment and the end date was the date of the last dispensation + the number of days supplied during the last dispensation.

A treatment break was considered to have occurred if a patient was not dispensed the treatment for a given time but re-initiated the same treatment later. Consecutive treatment episodes with the same treatment were merged if the treatment break between the current dispensing and the end of supply of the previous dispensing was within the allowable gap (i.e., grace period). The grace period used for all treatments was fixed for 60 days which was considered from date of end of supply of the last dispensation of the drug [15, 23]. For a given drug, a single patient could receive several dispensations of different durations in the supplied period.

#### Healthcare resource utilization and costs

HCRU and costs were assessed from the inclusion date, i.e., first diagnosis of PM/DM, to the end of follow-up or the end of the treatment. PM/DM-related HCRU was assessed as the number of inpatient and outpatient visits per patient/year; average duration of inpatient visits was also estimated per patient/year. PM/DM-related healthcare cost was defined as the total cost of all the PM/DM-related inpatient and outpatient visits, treatments, and procedures per patient/year.

### Statistical analyses

All the analyses were conducted from the primary study population, stratified by each treatment during the treatment initiation and by subgroup. The subgroups were based on pre-disease history: treatment-naïve and treatment-experienced, M33 code subcategories.

Frequencies and percentages were presented for categorical variables while continuous variables were summarized as number of observations (*n*), mean, standard deviation (SD), median, quartiles (Q; Q1; and Q3), minimum (min), and maximum (max) values. Sequence of treatment was graphically explored using Sankey-like plots after initiation of the first LoT. The distribution of prescriber’s specialty was analyzed for each treatment type and line. Chi-square test and linear regression model were used to assess the impact of patient characteristics on treatment choice.

## Results

### Patient characteristics

**Fig. 1:**
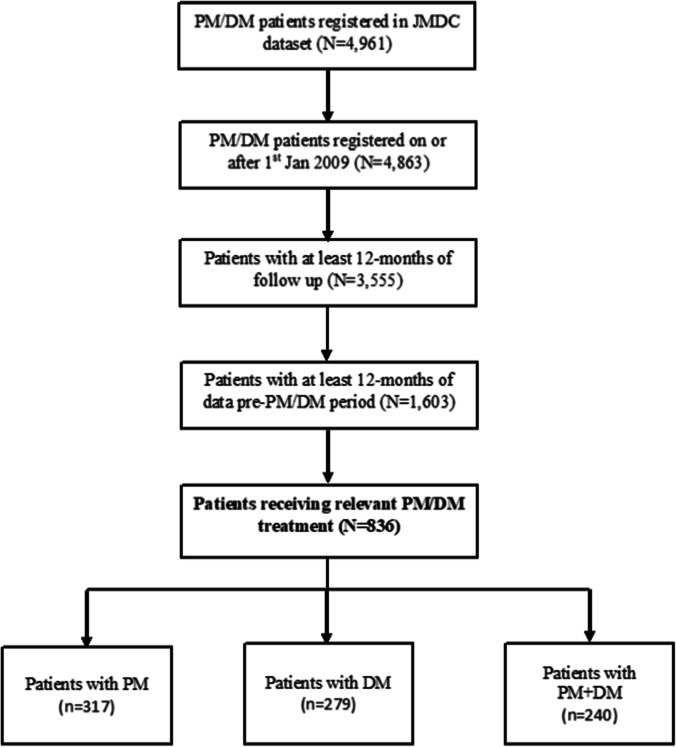
Patient characteristics. DM, dermatomyositis; JMDC, Japan Medical Data Center; PM, polymyositis; SD, standard deviation; Q, quartile. Note: all patients diagnosed inclusively with M33.0 (juvenile dermatomyositis), M33.1 (other dermatomyositis), M33.2 (polymyositis), and M33.9 (dermatomyositis, unspecified) were enrolled in the study. PM (M33.2)/DM (M33.1) is defined as the patient being diagnosed with PM first and later developed DM as well or vice versa

**Fig. 2:**
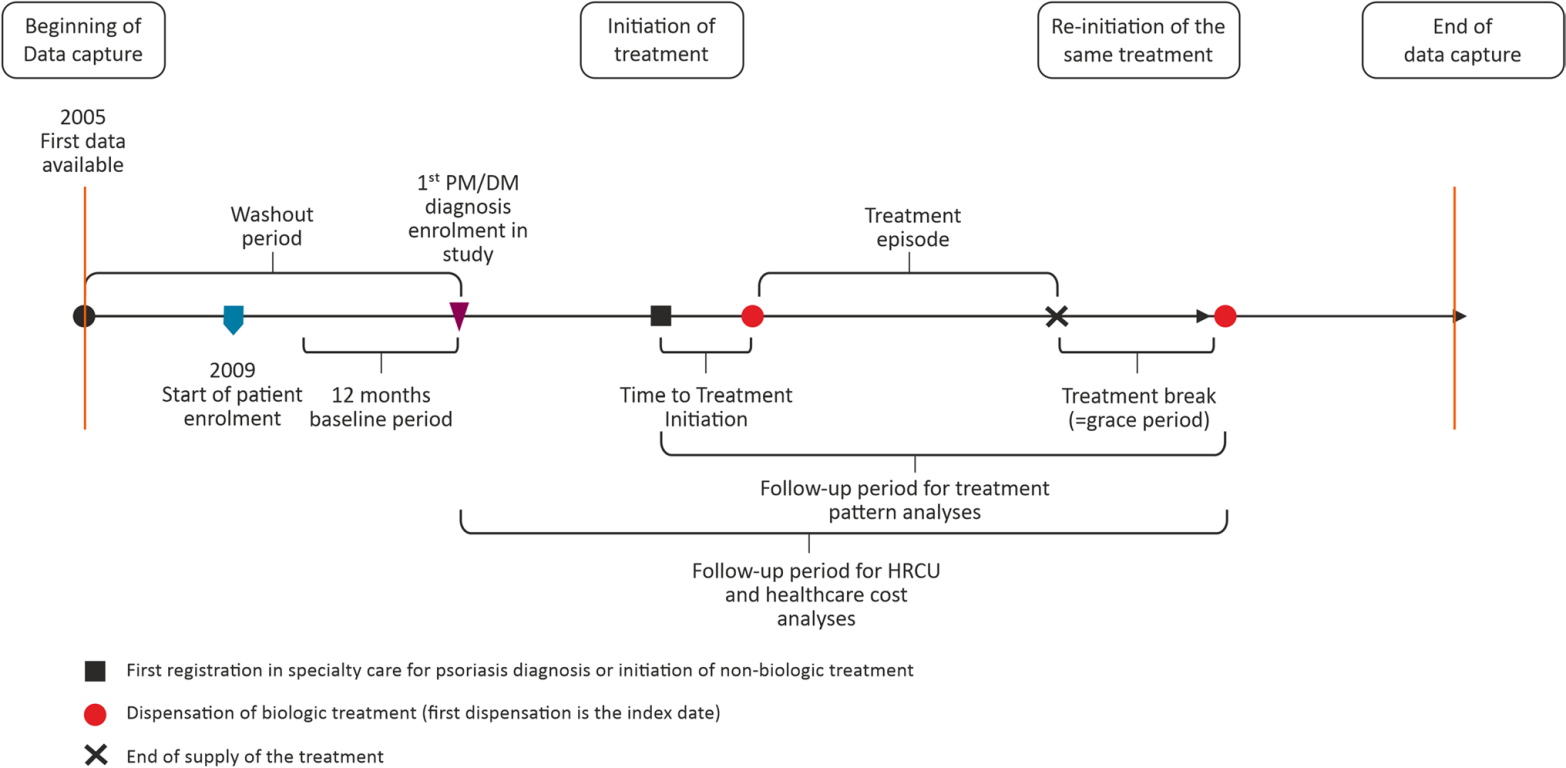
Study design and overview of treatment breaks. DM, dermatomyositis; HCRU, healthcare resource utilization; PM, polymyositis

A total of 4,961 patients with PM/DM characteristics were registered in the whole JMDC data set and 836 (52%) patients received a relevant treatment (Fig. 1), out of which the mean (SD) follow-up/patient recorded was 3.10 (1.90) visits. A total of 279/836 patients had a dislogistic record of DM, 317 patients had PM, and 240 patients had a dislogistic record for both PM and DM (PM + DM). The study design and treatment breaks are depicted in Fig. 2. Majority of the patients were female (60.4%). The overall mean (SD) age at first diagnosis was 46.75 (15.05) and at initiation of treatment was 46.94 (15.05). PM (66.6%) and DM unspecified (59.7%) were the major PM/DM subtypes identified (Table 1). Patient distribution by interstitial lung disease (ILD) (± other respiratory diseases), tumor/cardiovascular disease (CVD) at baseline, showed that majority of the patients belonged to the CVD subgroup (*n* = 222/836 [26.55%]) and ILD (± other respiratory diseases) ± tumor/CVD (*n* = 206/836 [24.64%]), while 42% (*n* = 348/836) had none of the above category. The highest frequency of PM/DM subtype recorded was in patients with ILD (± other respiratory diseases) + tumor + CVD (DM unspecified, 82.35% [*n* = 14/17]), CVD (PM, 72.97%, [*n* = 162/222]), tumor (PM and DM unspecified, 75.0% [*n* = 6/8], respectively), and tumor + CVD (PM, 77.14% [*n* = 27/35]) and 67.24%, *n* = 234/348 (PM) in patients belonging to none of the above category (Online Resource 1).

**Table 1:** Baseline characteristics of the patients

Category	Overall (*n* = 836)	Men (*n* = 331)	Women (*n* = 505)	PM (*n* = 317)	DM (*n* = 279)	PM + DM (*n* = 240)
Age at first diagnosis						
Mean (SD)	46.75 (15.05)	46.57 (15.67)	46.86 (14.64)	46.55 (14.39)	45.66 (16.37)	48.26 (14.35)
Median (Q1, Q3)	50 (39,58)	49 (38, 59)	50 (40, 57)	49 (39, 57)	49 (38, 58)	51 (43.5, 58)
Min, Max	1,74	1, 73	4, 74	3, 72	1,74	4,73
Age at initiation of treatment						
Mean (SD)	46.94 (15.05)	46.73 (15.65)	47.08 (14.66)	46.72 (14.40)	45.78 (16.26)	48.57 (14.33)
Median (Q1, Q3)	50 (39, 58)	50 (38, 59)	50 (40, 57)	50 (39, 58)	49 (38, 58)	51 (43.5, 58)
Min, Max	2, 75	2, 74	4, 75	4, 73	2, 74	5, 75
Sex, N (%)						
Men	331 (39.6)	331 (100)	0 (0)	137 (43.2)	108 (38.7)	86 (35.8)
Women	505 (60.4)	0 (0)	505 (100)	180 (56.8)	171 (61.3)	154 (64.2)
PM/DM subtype, N (%)						
Juvenile dermatomyositis	25 (3.0)	9 (2.7)	16 (3.2)	0 (0)	16 (5.7)	9 (3.8)
Other dermatomyositis	120 (14.4)	37 (11.2)	83 (16.4)	0 (0)	64 (22.9)	56 (23.3)
Polymyositis	557 (66.6)	223 (67.4)	334 (66.1)	317 (100)	0 (0)	240 (100)
Dermatomyositis, unspecified	499 (59.7)	188 (56.8)	311 (61.6)	0 (0)	267 (95.7)	232 (96.7)
Comorbidities, N (%)						
Malignant tumors	67 (8.01)	30 (9.06)	37 (7.33)	26 (8.2)	22 (7.89)	19 (7.92)
Acute cystitis	22 (2.63)	5 (1.51)	17 (3.37)	10 (3.15)	5 (1.79)	7 (2.92)
Anemia	181 (21.65)	58 (17.52)	123 (24.36)	85 (26.81)	58 (20.79)	38 (15.83)
Heart disease	293 (35.05)	114 (34.44)	179 (35.45)	119 (37.54)	95 (34.05)	79 (32.92)
Hypertension	197 (23.56)	89 (26.89)	108 (21.39)	77 (24.29)	67 (24.01)	53 (22.08)
Hypothyroidism	61 (7.3)	17 (5.14)	44 (8.71)	20 (6.31)	21 (7.53)	20 (8.33)
Osteoporosis	199 (23.8)	51 (15.41)	148 (29.31)	84 (26.5)	67 (24.01)	48 (20)
Temporal arteritis	0 (0)	0 (0)	0 (0)	0 (0)	0 (0)	0 (0)
Diabetes mellitus	217 (25.96)	96 (29)	121 (23.96)	88 (27.76)	73 (26.16)	56 (23.33)
Depression	69 (8.25)	22 (6.65)	47 (9.31)	38 (11.99)	19 (6.81)	12 (5)
Fibromyalgia	11 (1.32)	2 (0.6)	9 (1.78)	4 (1.26)	3 (1.08)	4 (1.67)
Respiratory diseases	226 (27.03)	80 (24.17)	146 (28.91)	41 (12.93)	97 (34.77)	88 (36.67)
ILD (± other respiratory diseases)	223 (26.67)	79 (23.86)	144 (28.51)	41 (12.93)	9 (3.22)	87 (36.25)
CCI score						
Mean (SD)	2.2 (1.8)	2.3 (2.0)	2.2 (1.7)	2.4 (2.0)	2.2 (1.6)	1.9 (1.7)
Median (Q1, Q3)	2 (1, 3)	2 (1, 3)	2 (1, 3)	2 (1, 3)	2 (1, 3)	2 (1, 2)
Min, Max	0, 14	0, 14	0, 14	0, 14	0, 10	0, 11

*CCI* Charlson comorbidity index; *DM* dermatomyositis; *ILD* interstitial lung disease; *PM* polymyositis; *SD* standard deviation; *Q* quartile

The overall mean (SD) CCI score was 2.2 (1.8) and heart disease (35.05%) was the most common comorbidity observed. The mean (SD) CCI score was lower in patients with PM + DM subtype (1.9 [1.7]) compared with only PM (2.4 [2.0]) and only DM (2.2 [1.6]) (Table 1). The highest mean (SD) CCI score was recorded for patients belonging to ILD (± other respiratory diseases) + tumor + CVD subgroup (6.2 [3.0]) and tumor subgroup (6.1 [4.1]) (Online Resource 2). Frequently identified comorbidities at the index dates in females (*n* = 505) compared with males (*n* = 331) were osteoporosis (148 [29.31%] vs 51 [15.41%]), anemia (123 [24.36%] vs 58 [17.52%]), and depression (47 [9.31%] vs 22 [6.65%], while hypertension was more frequent in males (89 [26.89%] vs 108 [21.39%]). Patients were recorded having heart disease (38%) in PM group, heart disease (34%), and respiratory disease (35%) in DM group and respiratory disease (37%) in PM + DM group (Table 1).

Among the type of treatments dispensed by prescriber’s specialties, highest frequency of dispensations was observed in specialty of general internal medicine (9,091 [18.4%]) followed by rheumatology (3,217 [6.5%]), respiratory (2,736 [5.5%]), neurology (1,596 [3.2%]), and dermatology (1,446 [2.9%]). The total number of dispensations was observed to be higher for systemic steroids (26,469 [100.0%]) and immunosuppressants (18,276 [100.0%]), also with the highest number of missing specialty data (14,479 [54.7%] and 11,250 [61.6%], respectively) (Online Resource 3).

### Treatment patterns and treatment choice

Japanese PM/DM patients were prescribed various treatments during the post-index period. The overall mean (SD) and median (Q1, Q3) time to initiation was 2.7 (7.3) and 0.8 (0.3, 1.6) months; time to switch was 11.3 (12.6) and 6.5 (3.0, 14.7) months; time to add-on was 7.7 (11.9) and 3.0 (1.9, 8.0) months; and time to discontinuation was 9.4 (15.2) and 3.0 (0.5, 11.8) months (Online Resource 4).

During the total of nine LoTs, the acute treatment episodes were not considered in the treatment pattern algorithm as the treatment duration was < 8 days (87/836 patients had only one acute treatment) and such episodes were observed separately. The total number of add-on and acute treatment episodes was 434 and 292, respectively. Frequently used add-ons were immunosuppressants (115/434 [26.5%]), topical steroids (104/434 [24.0%]), and non-steroidal anti-inflammatory drugs (NSAIDs) (103/434 [23.7%]). Additionally, frequently used acute treatments were NSAIDs (126/292 [43.2%]), systemic steroids (98/292 [33.6%]), and immunosuppressants (40/292 [13.7%]) (Online Resource 5).

Patients predominantly received first LoT (*n* = 749), second LoT (*n* = 292), and third LoT (*n* = 109). Patients received concomitant treatment during the first LoT (299/749 [39.9%]), second LoT (102/292 [34.9%]), and third LoT (37/109 [33.9%]). Patients also received systemic steroids (38/109 [34.9]) during third LoT (Online Resource 5).

A significant relation was observed on the type of comorbid condition and the choice of treatment (*P* < 0.0001). Patients receiving topical steroids (51 [17.35%]), systemic steroids (85 [28.91%]), immunosuppressants (9 [3.06%]), and NSAIDs (54 [18.37%]) were recorded having no comorbidity. Among patients who had ILD (± other respiratory diseases) alone/tumor + CVD as a comorbidity, 117 (57.64%) patients chose concomitant therapy. The patients with CVD chose either concomitant therapy (66 [33.5%]) or NSAIDs (60 [30.46%]), while patients who had CVD + tumor as the comorbidity chose either concomitant treatment (9 [30.03%]) or systemic steroids (9 [31.03]) as the choice of treatment (Table 2). The logistic regression results demonstrated the most probable treatment choice among patients. Males preferred systemic steroids over other treatments, while the patients with ILD (± other respiratory diseases) + tumor/CVD preferred combination therapy (OR [95% CI]: 2.544 [1.728–3.746]), DM patients preferred topical therapy (7.718 [4.187–14.227]), and PM + DM patients preferred combination therapy (2.464 [1.668–3.641]) (Table 2).

**Table 2:** Impact of characteristics on treatment choice

Categories	Concomitant		Topical steroids		Systemic steroids		Immunosuppressants		NSAIDs		*P* value
	*N* (%)	OR (95% CI)	*N* (%)	OR (95% CI)	*N* (%)	OR (95% CI)	*N* (%)	OR (95% CI)	*N* (%)	OR (95% CI)	
Age at treatment initiation											
0–20	22 (37.93)	Ref	11 (18.97)	Ref	13 (22.41)	Ref	1 (1.72)	Ref	11 (18.97)	Ref	0.2686
21–40	50 (36.76)	1.018 (0.527‒1.967)	18 (13.24)	0.859 (0.361‒2.044)	44 (32.35)	1.517 (0.735‒3.131)	1 (0.74)	0.39 (0.024‒6.426)	23 (16.91)	0.619 (0.263‒1.456)	
41–50	76 (39.79)	0.967 (0.513‒1.826)	29 (15.18)	1.261 (0.554‒2.870)	57 (29.84)	1.352 (0.667‒2.738)	4 (2.09)	1.238 (0.131‒11.664)	25 (13.09)	0.465 (0.199‒1.089)	
51–60	96 (40.34)	0.973 (0.522‒1.813)	36 (15.13)	1.239 (0.555‒2.766)	66 (27.73)	1.193 (0.594‒2.396)	12 (5.04)	3.205 (0.394‒26.075)	28 (11.76)	0.446 (0.193‒1.030)	
> 60	55 (44.0)	1.178 (0.597‒2.326)	15 (12.00)	0.86 (0.338‒2.185)	33 (26.40)	1.101 (0.514‒2.357)	9 (7.20)	4.564 (0.531‒39.197)	13 (10.40)	0.421 (0.161‒1.102)	
Sex											
Male	101 (35.44)	Ref	39 (13.68)	Ref	93 (32.63)	Ref	12 (4.21)	Ref	40 (14.04)	Ref	0.1974
Female	198 (42.76)	1.322 (0.96‒1.82)	70 (15.12)	1.148 (0.729‒1.808)	120 (25.92)	0.717 (0.516‒0.995)	15 (3.24)	0.786 (0.355‒1.740)	60 (12.96)	1.038 (0.654‒1.648)	
Comorbidities											
None	95 (32.31)	Ref	51 (17.35)	Ref	85 (28.91)	Ref	9 (3.06)	Ref	54 (18.37)	Ref	< 0.0001
ILD (± other respiratory diseases) + tumor/CVD)	117 (57.64)	2.544 (1.728‒3.746)	16 (7.88)	0.331 (0.177‒0.620)	52 (25.62)	0.906 (0.594‒1.381)	7 (3.45)	1.065 (0.37‒3.065)	11 (5.42)	0.355 (0.176‒0.72)	
Tumor	3 (37.5)	1.016 (0.226‒4.575)	2 (25.00)	1.932 (0.325‒11.479)	3 (37.50)	1.498 (0.342‒6.574)	0 (0.0)	NA	0 (0.0)	NA	
CVD	66 (33.5)	1.079 (0.723‒1.610)	30 (15.23)	0.98 (0.574‒1.675)	60 (30.46)	1.061 (0.705‒1.597)	9 (4.57)	1.024 (0.381‒2.755)	32 (16.24)	0.831 (0.494‒1.399)	
Tumor + CVD	9 (31.03)	1.009 (0.432‒2.357)	8 (27.59)	2.623 (0.998‒6.897)	9 (31.03)	1.106 (0.475‒2.575)	1 (3.45)	0.57 (0.066‒4.957)	2 (6.90)	0.27 (0.06‒1.221)	
ILD (± other respiratory diseases) + tumor + CVD	9 (52.94)	2.248 (0.813‒6.220)	2 (11.76)	0.395 (0.083‒1.886)	4 (23.53)	0.873 (0.268‒2.837)	1 (5.88)	1.154 (0.126‒10.553)	1 (5.88)	0.532 (0.065‒4.374)	
Disease type											
PM	70 (28.11)	Ref	15 (6.02)	Ref	83 (33.33)	Ref	14 (5.62)	Ref	67 (26.91)	Ref	< 0.0001
DM	105 (39.62)	1.441 (0.979‒2.122)	73 (27.55)	7.718 (4.187‒14.227)	57 (21.51)	0.574 (0.383‒0.861)	12 (4.53)	0.803 (0.347‒1.856)	18 (6.79)	0.190 (0.106‒0.340)	
PM + DM	124 (52.99)	2.464 (1.668‒3.641)	21 (8.97)	1.858 (0.919‒3.757)	73 (31.20)	0.967 (0.652‒1.433)	1 (0.43)	0.067 (0.009‒0.524)	15 (6.41)	0.197 (0.107‒0.363)	

Immunoglobulins were excluded from analysis since it was used only during first-line treatment

OR (95%CI) was calculated using logistic regression while the *P* values represent the results of Chi-square test

*CI* confidence interval; *CVD* cardiovascular disease; *DM* dermatomyositis; *ILD* interstitial lung disease; *NA* not available; *NSAIDs* non-steroidal anti-inflammatory drugs; *OR* odds ratio; *PM* polymyositis; *Ref* reference

### Healthcare resource utilization and cost

**Fig. 3:**
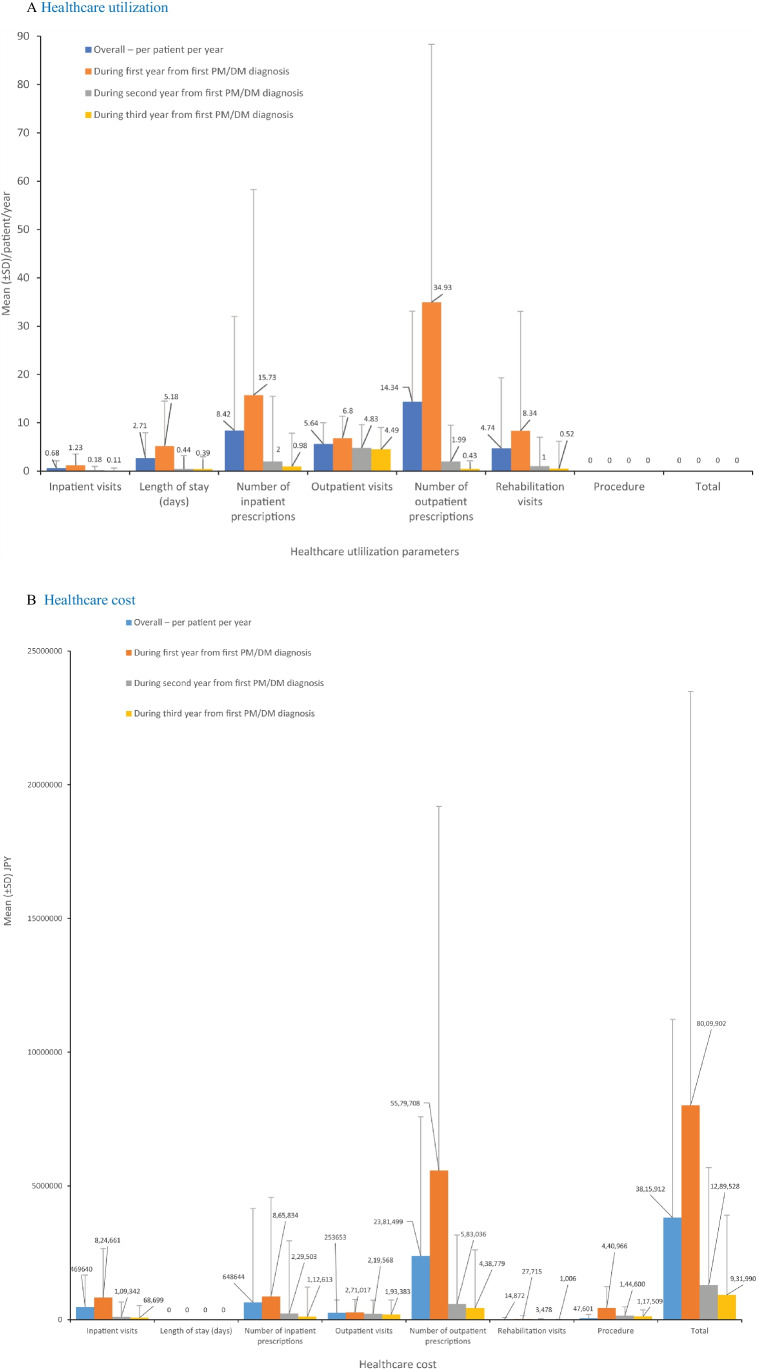
Healthcare utilization (**A**) and cost (**B**). Parameters during second year from first PM/DM diagnosis were calculated among the 558 patients with a follow-up of > 2 years. Parameters during third year from first PM/DM diagnosis were calculated among the 354 patients with a follow-up of > 3 years. DM, dermatomyositis; JPY, Japanese yen; PM, polymyositis; SD, standard deviation

HCRU during the first, second, and third year of PM/DM diagnosis is presented in Fig. 3A. The overall mean (SD) number of inpatient visits was observed to be very low 0.68 (1.43), length of stay was 2.71 (5.24) days, number of inpatient prescriptions was 8.42 (23.60), number of outpatient visits was 5.64 (4.36), number of outpatient prescriptions was 14.34 (18.79), and number of rehabilitation visits was 4.74 (14.57) per patient/year. A decreasing trend in HCRU was observed throughout the years for all the measurements (Fig. 3A). The number of mean (SD) dispensations per year was highest in systemic steroids (16.4 [20.5]) and immunosuppressants (17.9 [20.3]) (Online Resource 6). The overall mean (SD) healthcare cost per patient/year was ¥ 3,815,912 (7,412,241), with a decreasing trend observed throughout the years: first year (¥ 8,009,902 [15,473,597)], second year (¥ 1,289,528 [4,395,279]), and third year (¥ 931,990 [2,972,431]) (Fig. 3B).

## Discussion

This retrospective, observational real-world cohort study examined the characteristics of PM/DM patients and estimated their treatment patterns, prescription choice, and HCRU at healthcare setting in Japan. In our study, 60% were women, consistent to other evidence [17, 24, 25], and frequently observed baseline comorbidities were heart diseases, respiratory diseases, diabetes mellitus, and hypertension. Studies from Taiwan [26] and Canada [27] reported high prevalence of CVD and pulmonary diseases as baseline comorbidity. Other reports also suggested that comorbid complications develop in majority of patients at their first PM/DM diagnosis before initiating first LoT, mainly systemic steroid therapy. Therefore, clinicians and patients should increase their awareness of suitable medication regarding this risk [28]. Cardiac abnormalities are also reported to occur during any phase/after remission of PM/DM [29], increasing the risk of myocardial infarction [27]. ILD (27.03%) was also recorded at baseline in this claims database study and the odds of receiving concomitant treatment (systemic steroids + immunosuppressant) were doubled compared to other treatment groups, suggesting that patients were likely experiencing severe condition at first PM/DM diagnosis. Fujisawa et al. reported that myositis-associated acute ILD with older age is associated to poor prognosis in PM/DM patients [30]. Since ILD and heart disease are strongly correlated, considering an early referral to pulmonary specialists would be crucial [31]. Another baseline comorbidity observed was malignant tumor (8.01%), in patients with an average age of 46 years at first PM/DM diagnosis, and was found to be slightly higher in men (9.06%) than women (7.33%). Previous studies have suggested that men aged > 45 years are more often associated with developing a malignant tumor. Even though further evaluation is necessary to understand the correlation of these factors in PM/DM, CCI mean score of > 6 in patients with malignant tumor + ILD and CVD indicated higher risk of death in 1 year. Thus, patient awareness and implementing early-stage cancer screening can prove helpful towards clinical and public health in detecting cancer-associated myositis [32].

In our study, patients in first LoT preferred systemic steroids, followed by immunosuppressants + systemic steroids and in second LoT, patients preferred systemic steroids followed by immunosuppressants and then topical steroids. Overall, the patients took a median of 6 months to switch between treatments, suggesting that patients initiating treatment with steroids continued for a long time before switching, thus experiencing a burden (steroid myopathy affecting muscle recovery) due to steroid-related side effects. Thus, a high glucocorticoids (GC) dose is recommended to avoid myositis while keeping the treatment period as short as possible [8]. This burden could be the reason for the difference in the choice of treatment between two LoTs, which is also reported in previous studies that comorbidities do influence treatment choice and might also further complicate treatment operation for patients who switched multiple therapies in the past [26]. Other studies have also reported systemic corticosteroids as the most recommended initial therapeutic agent in PM/DM patients [8] while immunosuppressors being useful in inducing/maintaining remission/experiencing intolerable side effects with steroids in DM patients [33, 34]. Few studies have reported cyclosporin A (CsA) administration resulting in 75% reduction in the GC dose [35] and muscle recovery in JDM patients [8]. Our study established that switching within drug class was the most common action taken among patients and switches from the first to third LoT are mainly in between different concomitant therapies.

The results revealed that HCRU was higher for outpatient visits (5.64/patient/year) and rehabilitation (4.74/patient/year) relatively to hospitalization. The inpatient hospitalization (0.68/patient/year) was not high since PM/DM-related hospitalizations could not be linked to other comorbid diseases diagnosis when the event is not indicated for PM/DM. Therefore, we need to be mindful of the risks while managing the treatments. A study investigating the HCRU reported that PM/DM patients had 31.00% more medical visits, 44.00% more inpatient admissions, and 26.70% more outpatient/physician office visits than non-PM/DM patients [36]. Our study reported that among HCRU and cost burden, drug dispensation was frequent among all the cases, as there were many comorbid symptoms to be managed for PM/DM patients and their diagnosis changed by the time there was a necessity for a more intense care to be provided and thus the treatment was considered a non-PM/DM-related treatment. In this study, as per the prescriber’s specialties, general internal medicine had highest frequency of dispensations, since PM/DM symptoms often overlapped with other diseases, and thus could only be transferred to a specialist department after the general practitioner addressed the critical symptoms. Thus, the general practitioner must be mindful of such patients and refer them to appropriate specialists early for improved patient outcomes, and substantial decrease in healthcare cost [37]. PM/DM patients had a greater number of visits to rheumatologist, neurologist, and physical therapy and filled more prescriptions than non-PM/DM patients [36]. A study on commercial insurance data in the USA in 2016 reported that hospitalization for PM/DM is frequent (44%) with greater visits to a specialist vs general population (3.6 vs 2.5) [38]. The results demonstrated that the direct medical cost was mainly driven by outpatient (62%) drug cost (JPY 23,81,499) which is likely immunosuppressant + systemic steroids. The healthcare cost in studies conducted among the US population demonstrated that the PM/DM patients have comparatively more inpatients admissions, emergency department (ED) visits, and outpatient/physician visits, resulting in a medical care cost which was ~ 2.5 times ($12,145 vs $4,760) of the general control population [36]. Our study helped in building a more specific review on certain comorbidity in detail as it captures only the general PM, DM, and PM + DM with comorbidities like ILD (± other respiratory diseases), CVD, or tumor. Future studies will have to evaluate more patients particularly with comorbid conditions in interest.

Generally, RWE settings have multiple bias such as lack of follow-up, physician bias, recall bias, etc. Moreover, clinical trial settings have many patients excluded from the study, due to very strict classification criteria [39]. Doctors, clinicians, and patients consider randomized controlled trials for treatment decisions, but poor external validity (generalizability) leads to an underuse of few effective treatments, forcing them to make their judgements [40]. This further impacts real-world data to provide recommendations and hinders early diseases diagnosis/prognosis. Thus, documenting all the evidence is crucial in diagnosing diseases with complex characteristics—at early stage of development [41]. The study had few limitations; since a claims database was used, the data may have been affected by uneven coverage and update frequency of the database. The database may also not represent all the Japanese PM/DM patients as only 3% of the Japanese population are covered in the database. The retrospective nature of the study makes it prone to different types of bias such as sampling bias and confounding by changes in practice, as the impact of unmeasured confounders and risks on treatment decision cannot be identified by the researchers. However, these potential limitations are not expected to be a large issue in this disease area. Though we could categorize the groups as per the claims records, the patients’ severity in each group could not be identified, since the record of their condition description is unavailable in the database. We could not evaluate the clinical symptoms of PM/DM such as fever or fatigue and muscle symptoms of joint pains since the JMDC claims database is not specific to PM/DM. The data was also not specifically collected for the purpose of the study. There was no direct linkage observed between the dispensation and diagnosis, especially in patients who had multiple diagnosis. Since the study was conducted using data from insurance claims records, where clinical symptoms and progression of the patients are not collected, it was not possible to assess the reasons for initiating and discontinuation of a treatment. These limitations of the real-world data settings were taken into consideration while interpreting results of the performed analysis.

## Conclusion

This study is first to evaluate treatment pattern in the general cohort of PM/DM patients; very little is known about the preferred treatment pattern. Heart disease, lung disease, and diabetes were the frequently observed comorbidities in PM/DM patients. While systemic steroids were found to be the preferred treatment of choice in PM/DM patients, high concomitant immunosuppressant and systemic steroid prescriptions in the first LoT suggest that early optimal treatment is essential to manage PM/DM outcomes and disease cost burden in Japanese patients. However, a variety of specific autoantibodies have recently been identified in PM/DM, and the disease type classification has advanced. Therapeutic choice and establishment of disease management depending on the disease type are desired. For better disease management, it is necessary to further investigate these factors in the future.

### Electronic supplementary material

Below is the link to the electronic supplementary material.

Supplementary file1 (DOC 43 KB)

Supplementary file2 (DOC 47 KB)

Supplementary file3 (DOC 53 KB)

Supplementary file4 (DOC 40 KB)

Supplementary file5 (DOC 40 KB)

Supplementary file6 (DOC 31 KB)

## Data Availability

All relevant data are within the paper and its Supporting Information files. The data used for the current study are not publicly available because they were provided by Japan Medical Data Center (JMDC) to Analysis Group, Inc., and the data license agreement does not permit sharing of data sets with people external to the study team. Interested readers may request the data directly from JMDC.

## Appendix 1

**Table 3:** List of comorbidities

Comorbidity class	Comorbidity	ICD-10 code
Malignant tumors	Malignant tumors	All sub-codes of the C chapter
Acute cystitis	Acute cystitis	N30.0
Anemia	Aplastic and other anemias	All sub-codes of D60-D64
	Refractory anemia	D46.4, D46.5, D46.6, D46.7, D46.9
	Nutritional anemias	All sub-codes of D50-D53
	Hemolytic anemias	All sub-codes of D55-D59
	Anemia of prematurity	P61.2
	Other congenital anemias, not elsewhere classified	P61.4
Heart diseases	Hypertensive heart disease	All sub-codes of I11
	Hypertensive heart and renal disease	All sub-codes of I13
	Ischemic heart disease	All sub-codes of I20-I25
	Pulmonary heart disease	All sub-codes of I26-I28
	Other forms of heart disease	All sub-codes of I30-I52
Hypertension	Hypertensive diseases	All sub-codes of I10-I15
Hypothyroidism	Other hypothyroidism	All sub-codes of E03
Osteoporosis	Osteoporosis	All sub-codes of M81-M82
Temporal arteritis	Other giant cell arteritis	M31.6
Diabetes mellitus	Diabetes mellitus	All sub-codes of E10-E14
Depression	Depressive episode	All sub-codes of F32
	Recurrent depressive disorder	All sub-codes of F33
Fibromyalgia	Fibromyalgia	M79.7
Dermatomyositis	Juvenile dermatomyositis	All sub-codes of M330
	Interstitial pneumonia in juvenile dermatomyositis	
Other dermatomyositis	Respiratory disorder in dermatomyositis	All sub-codes of M331
	Interstitial pneumonia in dermatomyositis	
	Amyopathic dermatomyositis	
Polymyositis	Respiratory disorder in polymyositis	All sub-codes of M332
	Interstitial pneumonia in polymyositis	
	Polymyositis	

“PM” include patients that only had polymyositis during the follow-up (defined as polymyositis/M33.2). “DM” include patients that only had dermatomyositis during the follow-up (defined as juvenile dermatomyositis/M33.0, other dermatomyositis/M33.1 and dermatomyositis, unspecified/M33.9). PM + DM include patients that during follow-up had both polymyositis and dermatomyositis diagnose

## Appendix 2

**Table 4:** Study drugs

Drug subgroup	Drug name	ATC code
Topical steroids	Prednisolone	D07AA03
	Clobetasone	D07AB01
	Hydrocortisone butyrate	D07AB02
	Alclometasone	D07AB10
	Dexamethasone	D07AB19
	Betamethasone	D07AC01
	Fluocinolone acetonide	D07AC04
	Diflucortolone	D07AC06
	Fludroxycortide	D07AC07
	Fluocinonide	D07AC08
	Diflorasone	D07AC10
	Mometasone	D07AC13
	Beclometasone	D07AC15
	Difluprednate	D07AC19
	Clobetasol	D07AD01
	Tacrolimus	D11AH01
Systemic steroids	Fludrocortisone	H02AA02
	Betamethasone	H02AB01
	Dexamethasone	H02AB02
	Methylprednisolone	H02AB04
	Prednisolone	H02AB06
	Triamcinolone	H02AB08
	Hydrocortisone	H02AB09
	Cortisone	H02AB10
Immunosuppressive treatments	Sulfasalazine	A07EC01
	Cyclophosphamide	L01AA01
	Methotrexate	L01BA01
	Antithymocyte immunoglobulin (rabbit)	L04AA04
	Mycophenolic acid	L04AA06
	Leflunomide	L04AA13
	Abatacept	L04AA24
	Tofacitinib	L04AA29
	Etanercept	L04AB01
	Infliximab	L04AB02
	Adalimumab	L04AB04
	Certolizumab pegol	L04AB05
	Golimumab	L04AB06
	Basiliximab	L04AC02
	Ustekinumab	L04AC05
	Tocilizumab	L04AC07
	Ciclosporin	L04AD01
	Tacrolimus	L04AD02
	Azathioprine	L04AX01
	Methotrexate	L04AX03
	Lenalidomide	L04AX04
	Chlorambucil	L01AA02
	Rituximab	L01XC02
	Hydroxychloroquine	P01BA02
Intravenous immunoglobulin therapies	Immunoglobulins	All sub-codes of: J06B
Anti-rheumatics	Indometacin	M01AB01
	Sulindac	M01AB02
	Diclofenac	M01AB05
	Etodolac	M01AB08
	Acemetacin	M01AB11
	Lornoxicam	M01AC05
	Meloxicam	M01AC06
	Ibuprofen	M01AE01
	Naproxen	M01AE02
	Ketoprofen	M01AE03
	Flurbiprofen	M01AE09
	Tiaprofenic acid	M01AE11
	Mefenamic acid	M01AG01
	Flufenamic acid	M01AG03
	Celecoxib	M01AH01
	Other anti-inflammatory and anti-rheumatic agents, non-steroids	M01AX
