# Correction to: Disease burden and treatment sequence of polymyositis and dermatomyositis patients in Japan: a real-world evidence study

**DOI:** 10.1007/s10067-022-06242-8

**Published:** 2022-06-14

**Authors:** Celine Miyazaki, Yutaka Ishii, Natalia M. Stelmaszuk

**Affiliations:** 1 Health Economics Department, Janssen Pharmaceutical K.K., Tokyo, Japan; 2 Immunology, Infectious Diseases and Vaccine Department, Medical Affairs Division, Janssen Pharmaceutical K.K., Tokyo, Japan; 3 Real World Evidence Consultant, Parexel International, Stockholm, Sweden

**Correction to: Clinical Rheumatology (2021) 41:741–755**

**https://doi.org/10.1007/s10067-021-05939-6**

In the original published version of the above article, the co-author's given name was incorrectly spelled as "Yukata Ishii" instead of "Yutaka Ishii". The name is presented correctly above.

The original article has been corrected.

