# Supplementary material for: Disease burden and treatment sequence of polymyositis and dermatomyositis patients in Japan: a real-world evidence study
Source: Clin Rheumatol. 2021 Oct 22;41(3):741–55. doi: 10.1007/s10067-021-05939-6 (PMC8873135; doi:10.1007/s10067-021-05939-6)
Supplement: Supplementary file 1 — Supplementary file1 (DOC 43 KB) [file 10067_2021_5939_MOESM1_ESM.doc]

**Journal name:** Clinical Rheumatology

**Title:** Disease Burden and Treatment Sequence of Polymyositis and Dermatomyositis Patients in Japan: A Real-World Evidence Study

**Authors:** Celine Miyazaki^1^; Yukata Ishii^2^; Natalia M. Stelmaszuk^3^

**Affiliations:** ^1^Health Economics Department, Janssen Pharmaceutical K.K., Tokyo, Japan; ^2^Immunology, Infectious Diseases & Vaccine Department, Medical Affairs Division, Janssen Pharmaceutical K.K., Tokyo, Japan; ^3^ Real World Evidence Consultant, Parexel International, Sweden

**Corresponding author:** celinemiyazaki@gmail.com

**Online Resource 1 Distribution of PM/DM patients by ILD (±other respiratory diseases), malignant tumor and cardiovascular disease**

| **Category** | **ILD (±other respiratory diseases) + tumor + CVD (n=17)** | **CVD (n=222)** | **ILD (±other respiratory diseases) ± tumor/CVD (n=206)** | **Tumor (n=8)** | **Tumor + CVD (n=35)** | **None (n=348)** |
| --- | --- | --- | --- | --- | --- | --- |
| **Age at first diagnosis** | | | | |  |  |
| Mean (SD) | 56.71 (8.53) | 49.59 (13.20) | 50.17 (13.44) | 56.63 (11.88) | 55.20 (13.85) | 41.34 (15.70) |
| Median (Q1, Q3) | 58 (55, 61) | 51 (43, 59) | 52 (45, 59) | 56 (49.5, 66) | 59 (47, 65) | 45 (31, 53.5) |
| Min, Max | 41, 73 | 8, 73 | 1, 74 | 37, 73 | 9, 72 | 3, 72 |
| **Age at initiation of treatment** | | | | |  |  |
| Mean (SD) | 56.82 (8.48) | 49.79 (13.24) | 50.36 (13.42) | 56.88 (12.29) | 55.40 (13.92) | 41.53 (15.69) |
| Median (Q1, Q3) | 58 (56, 61) | 51 (43, 60) | 52 (45, 59) | 56 (49.5, 66) | 60 (49, 65) | 45 (32, 53) |
| Min, Max | 41, 73 | 10, 74 | 2, 74 | 37, 75 | 9, 73 | 4, 72 |
| **Sex** | | | | |  |  |
| Men, N (%) | 9 (52.9%) | 95 (42.8) | 70 (34.0%) | 3 (37.5%) | 15 (42.9%) | 139 (39.9%) |
| Women, N (%) | 8 (47.1%) | 127 (57.2%) | 136 (66.0%) | 5 (62.5%) | 20 (57.1%) | 209 (60.1%) |
| **PM/DM subtype, N (%)** | | | | |  |  |
| Juvenile DM | 0 (0%) | 3 (1.35%) | 4 (1.94%) | 0 (0%) | 1 (2.86%) | 17 (4.89%) |
| Other DM | 3 (17.65%) | 20 (9.01%) | 61 (29.61%) | 0 (0%) | 3 (8.57%) | 33 (9.48%) |
| PM | 7 (41.18%) | 162 (72.97%) | 121 (58.74%) | 6 (75%) | 27 (77.14%) | 234 (67.24%) |
| DM, unspecified | 14 (82.35%) | 112 (50.45%) | 159 (77.18%) | 6 (75%) | 14 (40%) | 194 (55.75%) |

CVD, cardiovascular disease; DM, dermatomyositis; ILD, interstitial lung disease; PM, polymyositis; SD, standard deviation; Q, quartile
