# Supplementary material for: Disease burden and treatment sequence of polymyositis and dermatomyositis patients in Japan: a real-world evidence study
Source: Clin Rheumatol. 2021 Oct 22;41(3):741–55. doi: 10.1007/s10067-021-05939-6 (PMC8873135; doi:10.1007/s10067-021-05939-6)
Supplement: Supplementary file 2 — Supplementary file2 (DOC 47 KB) [file 10067_2021_5939_MOESM2_ESM.doc]

**Journal name:** Clinical Rheumatology

**Title:** Disease Burden and Treatment Sequence of Polymyositis and Dermatomyositis Patients in Japan: A Real-World Evidence Study

**Authors:** Celine Miyazaki^1^; Yukata Ishii^2^; Natalia M. Stelmaszuk^3^

**Affiliations:** ^1^Health Economics Department, Janssen Pharmaceutical K.K., Tokyo, Japan; ^2^Immunology, Infectious Diseases & Vaccine Department, Medical Affairs Division, Janssen Pharmaceutical K.K., Tokyo, Japan; ^3^ Real World Evidence Consultant, Parexel International, Sweden

**Corresponding author:** celinemiyazaki@gmail.com

**Online Resource 2** Charlson’s comorbidity index for patients with ILD (±other respiratory diseases), tumor, CVD category


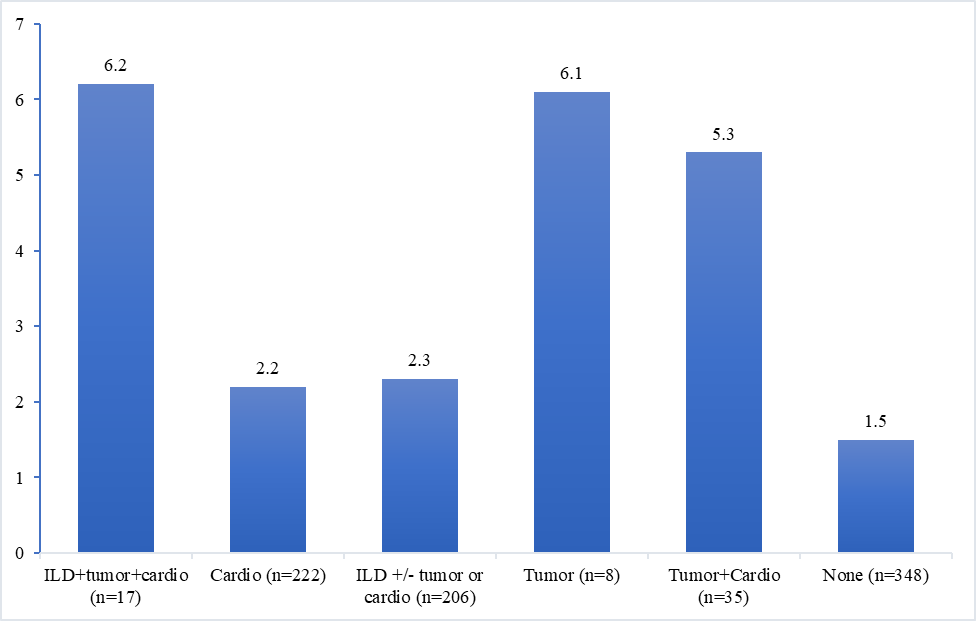


CVD, cardiovascular disease; ILD, interstitial lung disease
