# Supplementary material for: Disease burden and treatment sequence of polymyositis and dermatomyositis patients in Japan: a real-world evidence study
Source: Clin Rheumatol. 2021 Oct 22;41(3):741–55. doi: 10.1007/s10067-021-05939-6 (PMC8873135; doi:10.1007/s10067-021-05939-6)
Supplement: Supplementary file 3 — Supplementary file3 (DOC 53 KB) [file 10067_2021_5939_MOESM3_ESM.doc]

**Journal name:** Clinical Rheumatology

**Title:** Disease Burden and Treatment Sequence of Polymyositis and Dermatomyositis Patients in Japan: A Real-World Evidence Study

**Authors:** Celine Miyazaki^1^; Yukata Ishii^2^; Natalia M. Stelmaszuk^3^

**Affiliations:** ^1^Health Economics Department, Janssen Pharmaceutical K.K., Tokyo, Japan; ^2^Immunology, Infectious Diseases & Vaccine Department, Medical Affairs Division, Janssen Pharmaceutical K.K., Tokyo, Japan; ^3^ Real World Evidence Consultant, Parexel International, Sweden

**Corresponding author:** celinemiyazaki@gmail.com

**Online Resource 3** Frequency of treatment dispensations by prescriber’s specialty

| **Specialty, n (%)** | **Total dispensations** | **Immuno-globulins** | **Immuno- suppressants** | **NSAIDs** | **Systemic Steroids** | **Topical Steroids** |
| --- | --- | --- | --- | --- | --- | --- |
| Allergology | 275 (0.6) | 20 (3.1) | 20 (0.1) | 11 (0.5) | 224 (0.8) | 0 (0.0) |
| Cardiology | 258 (0.5) | 1 (0.2) | 109 (0.6) | 20 (0.9) | 128 (0.5) | 0 (0.0) |
| Dermatology | 1,446 (2.9) | 114 (17.6) | 409 (2.2) | 32 (1.4) | 718 (2.7) | 173 (9.3) |
| Gastroenterology | 168 (0.3) | 0 (0.0) | 15 (0.1) | 40 (1.8) | 111 (0.4) | 2 (0.1) |
| General Internal Medicine | 9,091 (18.4) | 212 (32.7) | 3,402 (18.6) | 225 (10.1) | 5,088 (19.2) | 164 (8.8) |
| Neurology | 42 (0.1) | 3 (0.5) | 8 (0.0) | 9 (0.4) | 21 (0.1) | 1 (0.1) |
| Neurology (IM) | 1596 (3.2) | 85 (13.1) | 221 (1.2) | 36 (1.6) | 1242 (4.7) | 12 (0.6) |
| Neurosurgery | 31 (0.1) | 0 (0.0) | 9 (0.0) | 2 (0.1) | 20 (0.1) | 0 (0.0) |
| Obstetrics & Gynecology | 348 (0.7) | 0 (0.0) | 23 (0.1) | 13 (0.6) | 250 (0.9) | 62 (3.3) |
| Ophthalmology | 10 (0.0) | 0 (0.0) | 1 (0.0) | 0 (0.0) | 9 (0.0) | 0 (0.0) |
| Orthopedics | 313 (0.6) | 0 (0.0) | 44 (0.2) | 79 (3.6) | 186 (0.7) | 4 (0.2) |
| Other Orthopedics | 27 (0.1) | 0 (0.0) | 0 (0.0) | 0 (0.0) | 9 (0.0) | 2 (0.1) |
| Other | 1 (0.0) | 0 (0.0) | 16 (0.1) | 0 (0.0) | 1 (0.0) | 0 (0.0) |
| Otorhinolaryngology | 3 (0.0) | 0 (0.0) | 0 (0.0) | 1 (0.0) | 2 (0.0) | 0 (0.0) |
| Pediatrics | 662 (1.3) | 31 (4.8) | 232 (1.3) | 15 (0.7) | 373 (1.4) | 11 (0.6) |
| Psychiatry | 375 (0.8) | 0 (0.0) | 123 (0.7) | 0 (0.0) | 252 (1.0) | 0 (0.0) |
| Radiology | 2 (0.0) | 0 (0.0) | 0 (0.0) | 0 (0.0) | 2 (0.0) | 0 (0.0) |
| Respirator Surgery | 10 (0.0) | 0 (0.0) | 0 (0.0) | 6 (0.3) | 2 (0.0) | 2 (0.1) |
| Respiratory | 2,736 (5.5) | 22 (3.4) | 1,144 (6.3) | 15 (0.7) | 1,473 (5.6) | 82 (4.4) |
| Rheumatology | 3,217 (6.5) | 103 (15.9) | 1,235 (6.8) | 104 (4.7) | 1,720 (6.5) | 55 (2.9) |
| Surgery | 212 (0.4) | 0 (0.0) | 12 (0.1) | 13 (0.6) | 156 (0.6) | 31 (1.7) |
| Urology | 7 (0.0) | 0 (0.0) | 3 (0.0) | 1 (0.0) | 3 (0.0) | 0 (0.0) |
| Missing | 28,652 (57.9) | 57 (8.8) | 11,250 (61.6) | 1,597 (72.0) | 14,479 (54.7) | 1,269 (67.9) |
| Total | 49,482 (100.0) | 648 (100.0) | 18,276 (100.0) | 2,219 (100.0) | 26,469 (100.0) | 1,870 (100.0) |

NSAID, nonsteroidal anti-inflammatory drug
