# Supplementary material for: Disease burden and treatment sequence of polymyositis and dermatomyositis patients in Japan: a real-world evidence study
Source: Clin Rheumatol. 2021 Oct 22;41(3):741–55. doi: 10.1007/s10067-021-05939-6 (PMC8873135; doi:10.1007/s10067-021-05939-6)
Supplement: Supplementary file 4 — Supplementary file4 (DOC 40 KB) [file 10067_2021_5939_MOESM4_ESM.doc]

**Journal name:** Clinical Rheumatology

**Title:** Disease Burden and Treatment Sequence of Polymyositis and Dermatomyositis Patients in Japan: A Real-World Evidence Study

**Authors:** Celine Miyazaki^1^; Yukata Ishii^2^; Natalia M. Stelmaszuk^3^

**Affiliations:** ^1^Health Economics Department, Janssen Pharmaceutical K.K., Tokyo, Japan; ^2^Immunology, Infectious Diseases & Vaccine Department, Medical Affairs Division, Janssen Pharmaceutical K.K., Tokyo, Japan; ^3^ Real World Evidence Consultant, Parexel International, Sweden

**Corresponding author:** celinemiyazaki@gmail.com

**Online Resource 4 Time to event among PM/DM patients**

| **Category (in months)** | **Overall**  **(N=836)** | **PM**  **(n=317)** | **DM**  **(n=297)** | **PM+DM**  **(n=240)** |
| --- | --- | --- | --- | --- |
| **Time to initiation** | | | | |
| Mean (SD) | 2.7 (7.3) | 2.2 (6.7) | 2.0 (5.0) | 4.3 (9.8) |
| Median (Q1, Q3) | 0.8 (0.3, 1.6) | 0.6 (0.3, 1.0) | 0.7 (0.4, 1.3) | 1.2 (0.6, 2.9) |
| Min, Max | 0; 104 | 0; 84.6 | 0; 63.7 | 0; 104.0 |
| **Time to switch** | | |  |  |
| Mean (SD) | 11.3 (12.6) | 11.9 (13.3) | 11.6 (12.2) | 10.6 (12.4) |
| Median (Q1, Q3) | 6.5 (3.0, 14.7) | 8.5 (3.0, 14.7) | 7.0 (3.3, 15.8) | 5.7 (3.0, 13.3) |
| Min, Max | 0.03; 76 | 0.03; 69.6 | 0.07; 76.2 | 0.03; 58.5 |
| **Time to add-on** | | |  |  |
| Mean (SD) | 7.7 (11.9) | 6.9 (8.1) | 7.8 (13.8) | 7.9 (12.1) |
| Median (Q1, Q3) | 3.0 (1.9, 8.0) | 3.9 (2.0, 10.1) | 2.9 (1.8, 5.7) | 3.0 (1.9, 8.8) |
| Min, Max | 0.06; 80.9 | 0.4; 40.9 | 0.07; 70.7 | 0.2; 80.9 |
| **Time to discontinuation** | | |  |  |
| Mean (SD) | 9.4 (15.2) | 7.2 (12.8) | 9.3 (14.2) | 11.4 (17.5) |
| Median (Q1, Q3) | 3.0 (0.5, 11.8) | 1.8 (0.2, 6.2) | 3.0 (1.0, 13.1) | 3.4 (1.0, 14.6) |
| Min, Max | 0; 123 | 0; 81.2 | 0; 113,0 | 0; 122.9 |

DM, dermatomyositis; PM, polymyositis; SD, standard deviation; Q, quartile
