# Supplementary material for: Disease burden and treatment sequence of polymyositis and dermatomyositis patients in Japan: a real-world evidence study
Source: Clin Rheumatol. 2021 Oct 22;41(3):741–55. doi: 10.1007/s10067-021-05939-6 (PMC8873135; doi:10.1007/s10067-021-05939-6)
Supplement: Supplementary file 5 — Supplementary file5 (DOC 40 KB) [file 10067_2021_5939_MOESM5_ESM.doc]

**Journal name:** Clinical Rheumatology

**Title:** Disease Burden and Treatment Sequence of Polymyositis and Dermatomyositis Patients in Japan: A Real-World Evidence Study

**Authors:** Celine Miyazaki^1^; Yukata Ishii^2^; Natalia M. Stelmaszuk^3^

**Affiliations:** ^1^Health Economics Department, Janssen Pharmaceutical K.K., Tokyo, Japan; ^2^Immunology, Infectious Diseases & Vaccine Department, Medical Affairs Division, Janssen Pharmaceutical K.K., Tokyo, Japan; ^3^ Real World Evidence Consultant, Parexel International, Sweden

**Corresponding author:** celinemiyazaki@gmail.com

**Online Resource 5 Treatment episodes by the line of treatment**

| **Treatment type, n (%)** | **Concomitant** | **Systemic steroids** | **Topical steroids** | **Immunosuppressants** | **Immunoglobulin** | **NSAIDs** | **Total** |
| --- | --- | --- | --- | --- | --- | --- | --- |
| Add-on treatment | 21 (4.8) | 44 (10.1) | 104 (24.0) | 115 (26.5) | 47 (10.8) | 103 (23.7) | 434 (100.0) |
| Acute treatment | 0 (0) | 98 (33.6) | 0 (0) | 40 (13.7) | 28 (9.6) | 126 (43.2) | 292 (100.0) |
| First-line | 299 (39.9) | 213 (28.4) | 109 (14.6) | 27 (3.6) | 1 (0.1) | 100 (13.4) | 749 (100.0) |
| Second-line | 102 (34.9) | 89 (30.5) | 33 (11.3) | 39 (13.4) | 0 (0.0) | 29 (9.9) | 292 (100.0) |
| Third-line | 37 (33.9) | 38 (34.9) | 16 (14.7) | 10 (9.2) | 0 (0.0) | 8 (7.3) | 109 (100.0) |
| Fourth-line | 6 (15.8) | 13 (34.2) | 4 (10.5) | 12 (31.6) | 0 (0.0) | 3 (7.9) | 38 (100.0) |
| Fifth-line | 6 (26.1) | 5 (21.7) | 6 (26.1) | 5 (21.7) | 0 (0.0) | 1 (4.3) | 23 (100.0) |
| Sixth-line | 1 (9.1) | 4 (36.4) | 2 (18.2) | 3 (27.3) | 0 (0.0) | 1 (9.1) | 11 (100.0) |
| Seventh-line | 1 (16.7) | 2 (33.3) | 1 (16.7) | 1 (16.7) | 0 (0.0) | 1 (16.7) | 6 (100.0) |
| Eight-line | 1 (25.0) | 1 (25.0) | 1 (25.0) | 0 (0.0) | 0 (0.0) | 1 (25.0) | 4 (100.0) |
| Ninth-line | 0 (0.0) | 1 (100.0) | 0 (0.0) | 0 (0.0) | 0 (0.0) | 0 (0.0) | 1 (100.0) |

NSAID, nonsteroidal anti-inflammatory drug
