# Supplementary material for: Disease burden and treatment sequence of polymyositis and dermatomyositis patients in Japan: a real-world evidence study
Source: Clin Rheumatol. 2021 Oct 22;41(3):741–55. doi: 10.1007/s10067-021-05939-6 (PMC8873135; doi:10.1007/s10067-021-05939-6)
Supplement: Supplementary file 6 — Supplementary file6 (DOC 31 KB) [file 10067_2021_5939_MOESM6_ESM.doc]

**Journal name:** Clinical Rheumatology

**Title:** Disease Burden and Treatment Sequence of Polymyositis and Dermatomyositis Patients in Japan: A Real-World Evidence Study

**Authors:** Celine Miyazaki^1^; Yukata Ishii^2^; Natalia M. Stelmaszuk^3^

**Affiliations:** ^1^Health Economics Department, Janssen Pharmaceutical K.K., Tokyo, Japan; ^2^Immunology, Infectious Diseases & Vaccine Department, Medical Affairs Division, Janssen Pharmaceutical K.K., Tokyo, Japan; ^3^ Real World Evidence Consultant, Parexel International, Sweden

**Corresponding author:** celinemiyazaki@gmail.com

**Online Resource 6** Average dispensation per year of follow-up

|  | **Systemic steroids** | **Topical steroids** | **Immunosuppressants** | **Immunoglobulin** | **NSAIDs** | **All PM/DM drugs** |
| --- | --- | --- | --- | --- | --- | --- |
| **Avg. dispensation per year of follow-up** | | | | | | |
| No. of patients, N (%) | 619 (74.0) | 267 (31.9) | 388 (46.4) | 57 (6.8) | 321 (38.4) | 836 (100.0) |
| Mean (SD) | 16.4 (20.5) | 2.9 (5.4) | 17.9 (20.3) | 5.6 (6.6) | 2.6 (5.1) | 22.8 (34.6) |
| Median (Q1, Q3) | 10.7 (2.2, 21.9) | 1.0 (0.5, 2.8) | 12.0 (4.8, 23.0) | 3.8 (1.5, 5.7) | 0.9 (0.4, 2.6) | 8.2 (1.3, 30.5) |
| Min, Max | 0.1, 175.0 | 0.1, 47.2 | 0.1, 141.5 | 0.1, 33.4 | 0.1, 49.1 | 0.1, 231.5 |

SD, standard deviation; Q, quartile
